# Estrogen fluctuations during the menopausal transition are a risk factor for depressive disorders

**DOI:** 10.1007/s43440-022-00444-2

**Published:** 2023-01-14

**Authors:** Justyna Turek, Łukasz Gąsior

**Affiliations:** Department of Neurobiology, Maj Institute of Pharmacology Polish Academy of Sciences, Smetna 12 Street, 31-343 Krakow, Poland

**Keywords:** Estrogen receptors, Menopause, Depression

## Abstract

Women are significantly more likely to develop depression than men. Fluctuations in the ovarian estrogen hormone levels are closely linked with women's well-being. This narrative review discusses the available knowledge on the role of estrogen in modulating brain function and the correlation between changes in estrogen levels and the development of depression. Equally discussed are the possible mechanisms underlying these effects, including the role of estrogen in modulating brain-derived neurotrophic factor activity, serotonin neurotransmission, as well as the induction of inflammatory response and changes in metabolic activity, are discussed.

## Introduction

Depression is the most common mental disorder with an ever-increasing occurrence. It is estimated that it affects about 10% of the human population. Although the average age for the development of depression is often between 30 and 40 years, the disease also occurs in children, adolescents, and the elderly [1, 2]. As the data show, boys in the prepubertal period are more likely than girls to be depressed. This trend is reversed in adolescence, and during the following years of life, women are about twice as likely as men to experience a depressive episode [3, 4].

Clinical and preclinical data have allowed scientists in depression research to formulate some hypotheses to explain the causes and development of the disease. One of the first, the “monoamine hypothesis,” assumed that depression is a result of lower concentrations of monoamines (serotonin, norepinephrine) in the brain structures responsible for emotional and cognitive processes (prefrontal cortex, PFC; hippocampus, Hip; amygdala, Amy) [5]. This conclusion was based on studies of the biological mechanisms of antidepressants (ADs), for which the therapeutic effects are associated with increased serotonergic and noradrenergic transmission through the inhibition of monoamine transporter function [selective serotonin reuptake inhibitors (SSRI) and noradrenaline (SNRI)], or inhibition of the enzymatic activity of the protein that catalyzes the breakdown of monoamines monoamine oxidase inhibitors (MAO).

A crucial biological phenomenon is associated with depression is stress. The reaction to stress, especially long term, is associated with a dysfunction of the hypothalamic–pituitary–adrenal axis (HPA). Hyperactivity of the HPA axis results in the increased release of glucocorticoids [6]. Interestingly, both clinical and preclinical studies have demonstrated increased activity of the HPA axis in depression and subsequent normalization of the same after long-term administration of antidepressants, including SSRIs. Moreover, the normalization of the HPA-axis activity has been correlated with improvements in mood, indicating that these effects may be related to the therapeutic actions of ADs [7]. Much attention has been focused on the role of stress and HPA-axis activation in the induction of brain-associated morphological changes in depression. Chronic stress causes atrophy of hippocampal pyramidal cells and inhibits neurogenesis in the cortex and Hip [8]. These morphological and functional changes in nerve cells are associated with the activation of the HPA axis, the concomitant release of excessive glucocorticoids, and damage of glutamate, serotonin, and dopamine neurons [8].

The activation of the HPA axis also leads to a reduction in the level of brain-derived neurotrophic factor (BDNF) [9], which promotes neuronal survival, growth, and plasticity of neural networks [10]. Reduced levels of BDNF induce Hip atrophy and reduction in Hip volume in patients with depression. On the other hand, ADs enhance the expression of BDNF, leading to the increased survival of neurons and the inhibition of stress-induced neurodegenerative processes. This confirms that the important role BDNF plays in the mechanism of antidepressant action and the adaptive changes that follow. These changes relate to the processes of transcription and translation of proteins involved in synaptic plasticity. One of the most important cellular responses to ADs, including SSRIs, is the increased expression of the transcription factor CREB (cyclic AMP-responsive element binding) in the Hip. CREB regulates the gene expression of synaptic plasticity and trophic factors for neurons, including BDNF [10]. BDNF and neurotrophin-3 (NT-3) stimulate serotonin and norepinephrine neuronal function resulting in the increased metabolism of serotonin and norepinephrine in the Hip [11].

The adverse effects of chronic stress and glucocorticoids on neurons may also be associated with the increased release of glutamate, resulting in the excitotoxic neuronal phenomenon, in which the substrate is the glutamate *N*-methyl-d-aspartate receptor (NMDA) [12]. Confirmation of this hypothesis came from the normalization of increased glucocorticoids and extracellular glutamate levels following the administration of NMDA receptor antagonists [13]. Moreover, many studies have shown both increased and decreased levels of glutamate in the serum of patients with depression and fluctuations in the concentration of glutamate in the PFC [14].

A growing body of evidence indicates that depression is accompanied by the activation of inflammatory and cell-mediated immune pathways, and increased oxidative and nitrosative stress (O&NS) [15]. The activation of the inflammatory and neurodegenerative pathways leads to brain damage observed in depression via both reduced neurogenesis and increased neurodegeneration [16]. On the other hand, cytokine-targeted drugs are effective in treating depression [17].

Recent clinical and preclinical studies suggested also that alterations in mitochondrial functions may precede the development of depression [18].

Evidence abounds that some of the changes mentioned above may be a consequence of alterations in the level of sex hormones, especially estrogen [19–22]. Furthermore, fluctuations in estradiol during the perimenopausal period are known to increase the sensitivity of women to psychosocial stress [23], which in turn is considered a phenomenon that primes the development of depressive disorders and is a widely recognized model of inducing depressive-like behaviors in rodents [24]. Epidemiological studies show that women are twice as likely to develop depression as men, which is probably related to dynamic fluctuations in sex hormones compared to men, whose sex hormones do not fluctuate and remain relatively constant throughout their lives [25, 26]. The occurrence of depression correlates with significant changes in blood estradiol levels in females, i.e., the period before menstruation (the so-called premenstrual dysphoric disorder, or PMDD) [27], the postpartum period (the so-called postpartum depression) [28], and the perimenopausal period [29]. Peri-menopausal women who have not had a previous episode of depression may experience depressed mood two-to-four times more often compared to pre-menopausal women. This risk is even higher in women with a history of depression [30–35].

Premature ovarian failure (primary ovarian insufficiency, POW), described as the loss of ovarian activity before age 40, affects approximately 1% of the population [36]. POW studies show that women with this disease are more likely to be depressed (54.5%) compared to the general population (20%). Some studies also indicate a significantly increased risk of depression after ovariectomy [37]. An increased risk of depression was also observed in perimenopause with naturally occurring menopause [29], which may be associated with increased variability in estradiol levels [38, 39]. This relationship is indicated by direct estradiol studies showing that pharmacological manipulation of estrogen levels in women resulted in depressive symptoms compared to the placebo group [40]. Clinical studies have shown, however, that estradiol can be considered an effective treatment for depression in perimenopausal [41] but not in post-menopausal women [42], further suggesting that fluctuations in estradiol levels (rather than absolute levels of estrogen) could play a role for both the development of depression in midlife women (window of vulnerability) and the potential antidepressant effects of estrogen in a particular population at a particular time (window of opportunity). The reason for this, however, is unknown [43].

This narrative review discusses research findings on estradiol function in the nervous system and the potential relationship between the development of depression and hormonal alteration during the perimenopausal period. The cited data come from publications published mainly in the last 20 years.

## Menopause

Currently, there is an upward trend in the number of menopausal women. By 2030, the global population of perimenopausal women will be about 1.2 billion, with an annual increase of 47 million [44]. The conventionally accepted age of 50 is somewhat an indicator of the onset of menopause. This critical stage of aging is associated with changes in the body, including changes in lipid profile and neurodegeneration [45]. Biological changes that accompany menopause might result in Alzheimer's disease (AD), Parkinson's disease (PD), depression, and cardiovascular disease [46, 47].

**Fig. 1:**
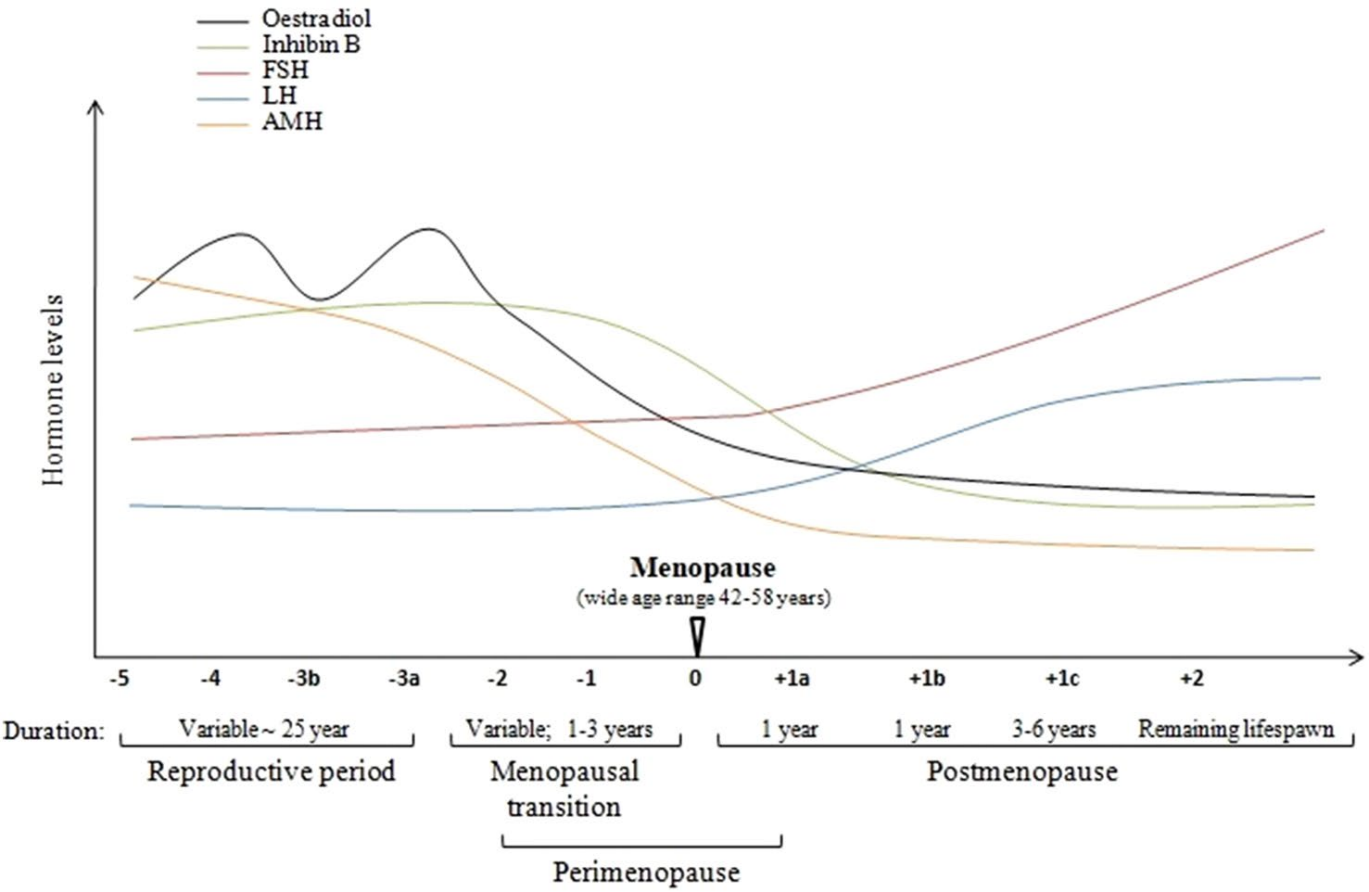
Stages of aging of the reproductive system in women. STRAW (Stages of Reproductive Aging Workshop) comprises ten stages beginning with the onset of menstrual cycles: reproductive age (− 5: early; − 4: peak fertility; − 3b − 3a: late reproductive stage), perimenopause (− 2 − 1: early and late menopausal transition), and postmenopause (+ 1a + 1b + 1c:early stages and + 2 late stage). With the onset of menopause, ovaries with fewer follicles induce a decrease in AMH (anti-Müllerian hormone), estradiol, and inhibin B production. The lack of ovarian responsiveness to FSH (follicle-stimulating hormone) and LH (luteinizing hormone) contributes to a negative feedback loop for estradiol and inhibin B resulting in the increased hypothalamic–pituitary release of GnRH (gonadotropin-releasing hormone) and FSH, and LH. Elevated FSH levels are particularly characteristic of the post-menopausal period. Note. The data are from Executive Summary of the Stages of Reproductive Aging Workshop 10: Addressing the Unfinished Agenda of Staging Reproductive Aging, by Harlow et al. [50]

The World Health Organization (WHO) defines natural menopause as the permanent cessation of the menstrual cycle resulting from the loss of ovarian follicle function. Entrance into menopause is considered a condition in which no menstrual bleeding has occurred for 12 consecutive months, not including other physiological or pathological causes (Report of a WHO Scientific Group [48]). This definition includes premenopause, perimenopause, menopausal transition, and overlapping menopause. The lack of standardized criteria to describe the stages of reproductive aging in women led to the creation of the Stages of Reproductive Aging Workshop (STRAW) in 2001. STRAW considers the frequency of menstrual cycles, endocrine factors, biochemical factors, and symptoms of other organ systems. Accordingly, STRAW split menopause into six pre-menopausal and four post-menopausal stages, referred to as the reproductive period, menopausal transition, menopause, and postmenopause (Fig. 1) [49, 50].

**Fig. 2:**
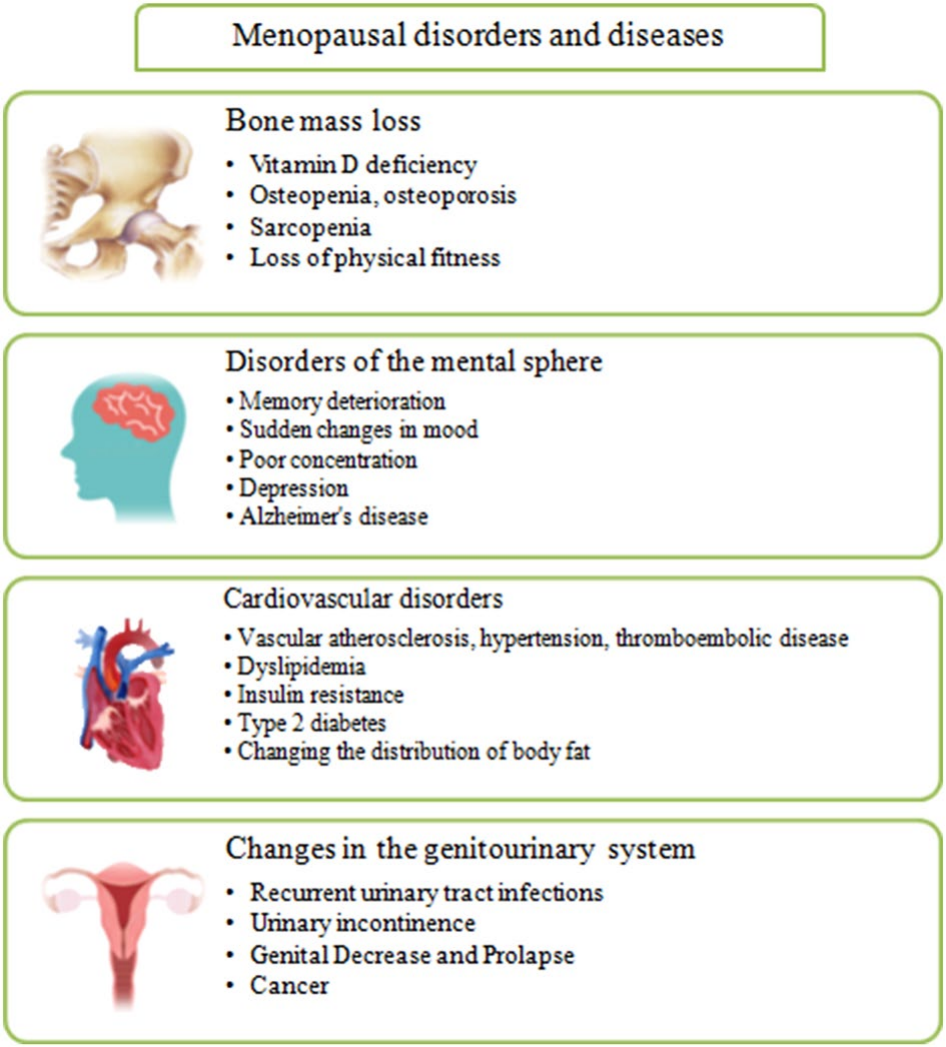
Hormonal changes occurring in the perimenopausal period are responsible for many ailments and adversely affect the quality of life of women. The substrate of these disorders changes ovarian function [decreased maturation of Graff follicles, insufficiency of corpus luteum, deficiency of progesterone, estrogen, ovarian inhibin, and increased concentration of the FSH (follicle-stimulating hormone)]

It is currently difficult to accurately predict the onset of menopause, especially in the preceding stage of the menopausal transition. Such knowledge would allow earlier intervention to alleviate the symptoms of aging in women. The period between menopausal transition and menopause is seen as a time of increased risk for AD [51]. The decreased production of ovarian estrogen during menopause leads to changes in connective tissues, metabolic disorders, cardiovascular diseases, genitourinary complaints, sleep disorders, and mood changes, including depressive disorders such as sudden mood changes, fatigue, nervousness, memory deterioration, and difficulties in coping with stressful situations (Fig. 2) [52]. It should be noted that there are individual variations in response to menopause. Factors influencing the menopausal period include the age at which menopausal changes occur, health status, environment, and lifestyle.

## The hypothalamic–pituitary–gonadal axis

The female reproductive cycle involves the hypothalamic–pituitary–ovarian axis activation, which controls the cyclic maturation of ovarian follicles. At the beginning of each cycle, the secretion of gonadotropin-releasing hormone (GnRH) from the hypothalamus stimulates the pulsatile secretion of follicle-stimulating hormone (FSH) and luteinizing hormone (LH) in the pituitary gland. These in turn act on specific ovarian receptors leading to the development of the ovarian follicle, oocyte maturation, and the production of estrogens by the follicular cells. In the early stages of this process, estrogen production is low. When the ovarian follicle is mature, there is a surge in the production of estradiol which leads to an increase in LH secretion (positive feedback), the swelling of the follicle, and the release of the oocyte into the fallopian tube. The luteal phase then follows ovulation. The follicle becomes a corpus luteum producing estradiol, progesterone, and inhibin, which inhibits LH and FSH secretion from the pituitary and GnRH from the hypothalamus. The corpus luteum disappears after about 14 days if fertilization does not occur, resulting in a decrease in estrogen, progesterone, and inhibin, and subsequent GnRH release from the hypothalamus to start the cycle again [53]. During the perimenopausal period, the ovarian follicle pool is depleted, decreasing steroid hormone and inhibin production. As a result, the loss of negative feedback leads to increased levels of the hypothalamic hormone GnRH and pituitary-derived LH and FSH (Fig. 1) [54].

## Estrogen receptors

**Fig. 3:**
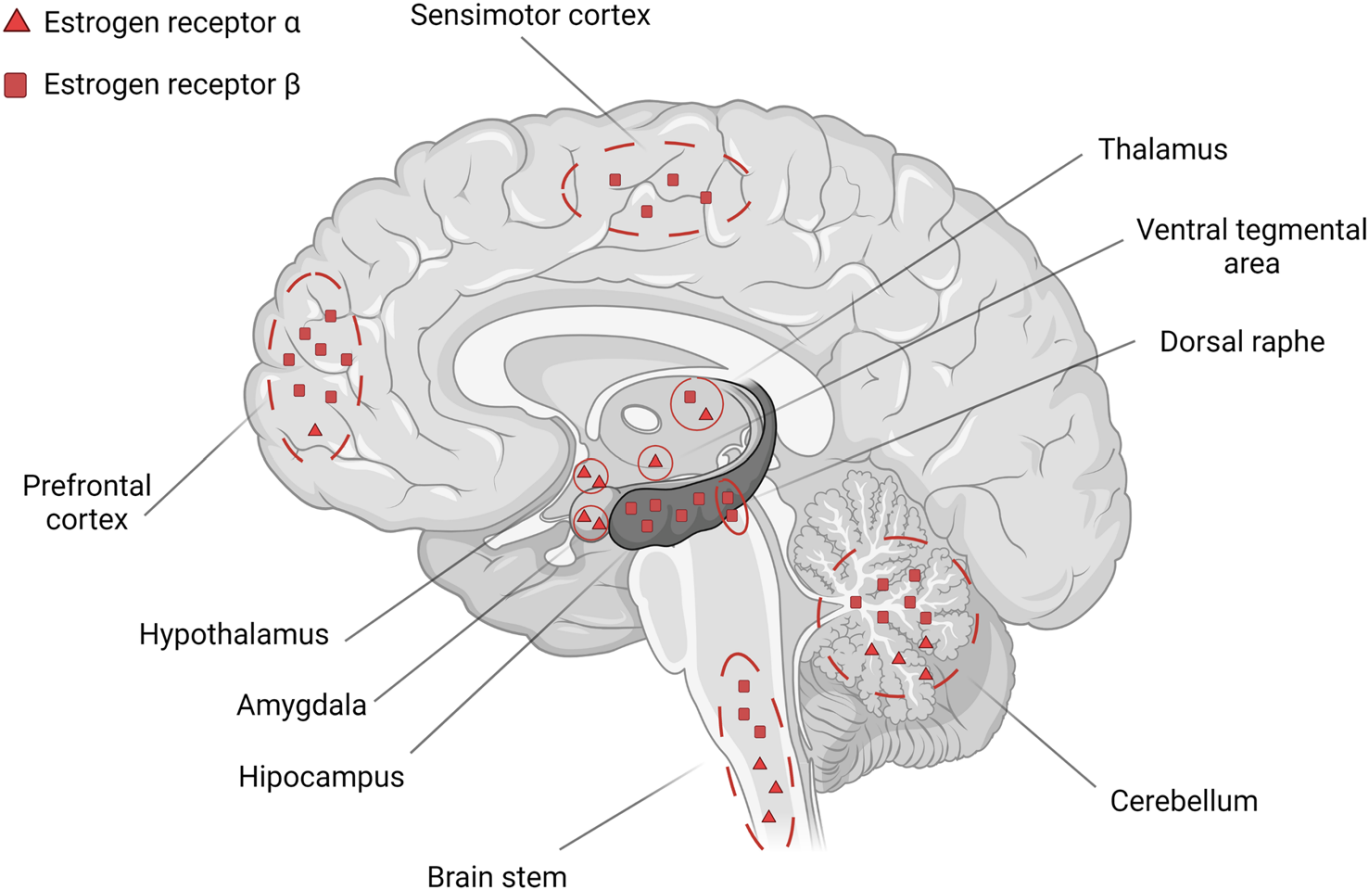
ER (estrogen receptors) in the brain. Most receptors are located in the Cb (cerebellum), PFC (prefrontal cortex), Hip (hippocampus), and Amy (amygdala). ERα is located in the Amy and hypothalamus, whereas ERβ is found mainly in the Hip. In the Cb and thalamus, both receptors are expressed simultaneously. Created with BioRender.com

**Fig. 4:**
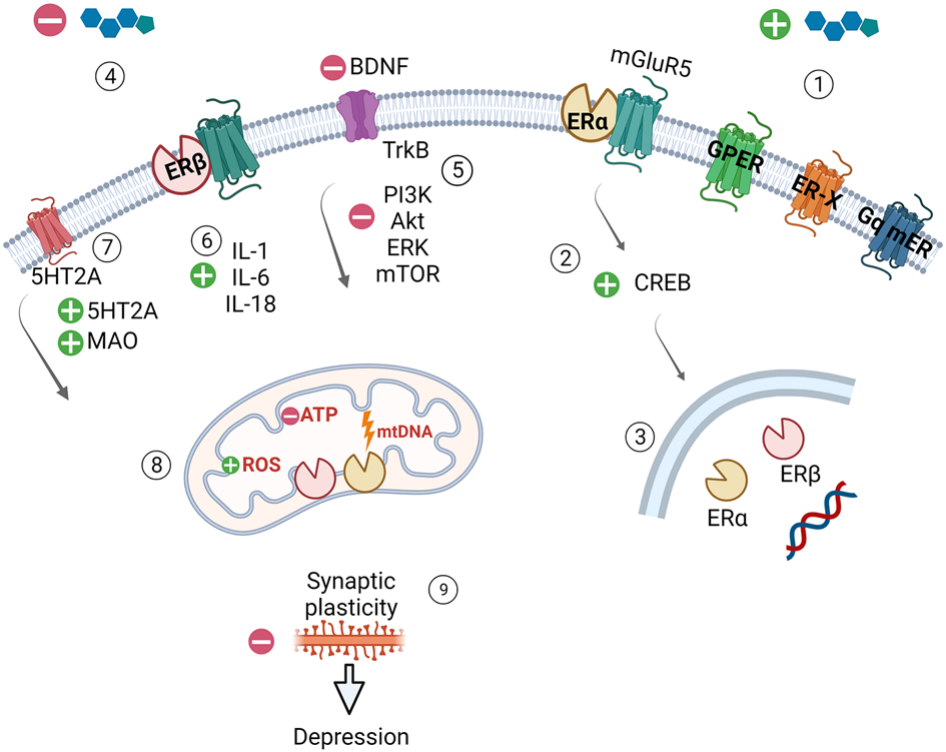
Simplified model showing estrogen receptor signaling in the Hip (hippocampus). E2 (estradiol) influences CNS (Central Nervous System) function through modulation of gene transcription (3) and rapid membrane signaling (1, 2). E2 by binding to the membrane ER (estrogen receptors) (4), rapidly activates signaling cascades involved in synaptic plasticity processes, thereby affecting memory processes. Membrane receptors ERα and ERβ lack the intrinsic ability to activate secondary messenger systems but bind to other membrane receptors mGluR (the metabotropic glutamate receptors). In Hip neurons, membrane receptor ERα activates mGluR5 and subsequently increases activation of the cellular transcription factor CREB (cAMP response element-binding protein) (2). Estrogens also activate membrane receptor subtypes: GPER-1 (G protein-coupled estrogen receptor), Gq-mER (Gq-coupled membrane estrogen receptor), and ER-X (1). Membrane receptors initiate Ca^2+^ signaling and activate PLC (phospholipase C) and AC (adenylyl cyclase), followed by the activation of multiple kinases: PI3K (phosphoinositide 3-kinase), ERK, AKT (protein kinase B), PKA (protein kinase A), PKC protein kinase C (5) which in turn can rapidly affect neuronal physiology by phosphorylation of transcription factors such as CREB or ERα to induce gene transcription. The relationship between serotonin and BDNF (brain-derived neurotrophic factor) TrkB (tropomyosin receptor kinase B) signaling is regulated by ERβ signaling in the hippocampus. Decreased ERβ receptor activity (4) decreases BDNF levels (5) and increases 5-HT2A and MAO (monoamine oxidase) levels (7). Aging is associated with altered brain bioenergetics, leading to reduced ATP (adenosine triphosphate) levels, decreased antioxidant defense, and increased ROS production and mtDNA (mitochondrial DNA) damage (8). Estrogen deficiency exacerbates immune dysfunction by activating the pro-inflammatory cytokines IL-1, IL-6, and IL-18 (6). Depressive disorders are caused by neuroanatomical changes involving the limbic system, including the Hip. Changes in synaptic transmission due to decreased estrogen production and neurotransmitter transmission contribute to reduced synaptic plasticity. Synaptic loss and deficits in functional connectivity include alterations in the AMPA (α-amino-3-hydroxy-5-methyl-4-isoxazolepropionic acid) receptor and NMDA (*N*-methyl-d-aspartate) receptor function, changes in glutamate release, and structural alterations of dendritic spines (9). Created with BioRender.com

Estrogen is the primary female sex hormone. The estrogen hormone family includes estrone, estradiol (E2, 17β-estradiol), and estriol. 17β-estradiol is the predominant form of estrogen, synthesized mainly by the ovaries, can cross the blood–brain barrier, or can be synthesized in the central and peripheral nervous systems by neurons and glial cells [55]. Estrogen exerts its action through estrogen receptors (ER) expressed in reproductive tissues, metabolic tissues (kidney, liver, and white adipose tissue), the lungs, bladder, and gastrointestinal tract [56]. In the brain, ERs are located in structures such as the PFC, Hip, Amy, and cerebellum (Cb), structures that are involved in learning and memory (Fig. 3) [57]. Two estrogen receptor subtypes (ERα and ERβ) are present in the human and rodent brains (Fig. 3) [58]. These receptors are located to the membrane, nuclear, and cytoplasmic and directly stimulate non-genomic and genomic pathways in neurons (Fig. 4) [59]. Nuclear receptors (ERα, ERβ) activated by steroid sex hormones, including estradiol, show slower regulation. Genomic regulation triggers gene transcriptions over a more extended time. In contrast, the non-genomic pathway, which activates membrane and cytoplasmic receptors (GPER, Gq, ERα, ERβ, ER-X), occurs within seconds [60].

## Estrogen and BDNF

As mentioned above, activating genomic and non-genomic pathways related to estrogen receptors leads to the activation of transcription factors and, consequently, to a change in gene expression in trophic factor genes, such as BDNF and glial cell-derived neurotrophic factor (GDNF) [61]. BDNF belongs to the family of neuronal growth factors and is secreted by neurons [62]. It determines neuronal survival and differentiation during development [62]. In addition, BDNF regulates excitatory and inhibitory synaptic transmission, learning and memory processes in the adult brain [63–65]. It is one of the most studied depression markers with low levels found in the PFC, Hip, and plasma of depression patients [66]. Furthermore, reduced expression of BDNF has been found in the postmortem brain of suicide subjects [67–69]. Interestingly, antidepressants are known to restore BDNF levels in the plasma of depressed patients [70].

Previous studies indicated that E2 and BDNF activate similar signaling pathways in the Hip, and E2 directly increases the expression of BDNF [20, 71]. Furthermore, BDNF expression fluctuations follow E2 level changes during the menstrual cycle [72]. Ovariectomy is a well-established method for E2 deprivation in animal models [73, 74]. Recent studies have indicated that the reduced level of E2 after ovariectomy results in decreased expression of BDNF in the Hip and PFC in rodents (Fig. 4) [73, 74]. At the same time, E2 supplementation in such animals reverses the BDNF down-regulation and prevents the development of a depression-like phenotype [75, 76]. These data suggest that menopause-related E2 deprivation and the associated decrease in BDNF expression may be one of the mechanisms underlining the increased risk of depression in menopausal women [77].

## Estrogen, memory, and cognitive impairments in perimenopause

During menopause, many organs, including the brain, are involved in the process of aging. The disruption of the hypothalamic–pituitary–gonadal axis in the pre- and post-menopausal periods also induces neurodegenerative processes. The dynamics of this transformation include alterations in brain structure (disturbances in synaptogenesis, changes in the number and morphology of neurons) and modifications in the bioenergetics of the brain, including glucose metabolism, mitochondrial respiration, and ATP (adenosine triphosphate) synthesis [78]. Due to disturbances in neurosteroid production, including estrogens, many CNS (central nervous system) processes related to the Hip, such as memory and cognition, deteriorate [79]. Women in the perimenopausal period have problems with memory, forgetfulness, and trouble finding the right words during conversations [80]. Memory impairment and the development of dementia are believed to be related to abnormalities in the formation of new neurons in the Hip [81, 82]. Previous studies have shown that hippocampal neurogenesis strongly depends on E2 activity in the Hip [83]. Interestingly, aging-related cognitive dysfunction progresses faster in women than in men [84].

Furthermore, E2 levels appear to be higher in older men than in post-menopausal females [85]. This positive correlation between neurogenesis and E2 level has been shown in several studies and the fact that E2 administration increases neural stem cell proliferation in vitro and improves cognition in physiological and pathological states in women [86]. At the same time, there is no significant effect of E2 on neurogenesis in the male Hip [87]. It should be noted that neurogenesis, the formation of dendritic spines, and new connections between neurons also depend on estradiol levels. For example, the density of dendritic spines in the rodent Hip changes throughout the menstrual cycle, with the highest density observed when E2 reaches its highest peak [88].

Likewise, after ovariectomy, rodents exhibit a lower density of dendritic spines in the Hip and PFC [89]. Similar observations have been made in humans. Dendritic spine number and Hip volume tend to be very high in the late follicular phase, i.e., a period of increased E2 production [90, 91]. There is also documented evidence of increased Hip volume after estrogen supplementation in post-menopausal women [92].

The formation of new dendritic spines is believed to be associated with the activation of the membrane estrogen receptor ERβ [93]. Selective ERβ agonists increase levels of hippocampal synaptic proteins [94] and are potent in improving cognitive abilities after ovariectomy [95]. It is generally believed that the number, size, and shape of dendritic spines are crucial for synaptic plasticity and thus learning and memory [96]. One of the most pronounced structural changes in the brain of depressed patients is reduced Hip volume [97] and depressed patients are twice as likely to develop dementia [98]. E2 levels affect the formation of new neurons and dendritic spines, crucial for neuroplasticity and learning processes. Therefore, impaired neuroplasticity due to reduced E2 levels may explain the increased risk for depressive disorders during the perimenopausal period in women.

## Estrogen and serotonin

Estrogen dramatically affects the levels of monoaminergic neurotransmitters in the brain. Serotonin (5-HT) plays a crucial role in the monoamine hypothesis of depression [99]. Increased bioavailability of 5-HT reduces depressive symptoms. The coexistence of receptors for estrogen and 5-HT in many tissues suggests mutual interaction between these receptors. Changes in estrogen levels affect 5-HT levels in two ways. First, increased synthesis of E2 increases the level of tryptophan hydroxylase (the biosynthetic enzyme for serotonin) and a decrease in serotonin catabolism through a reduction in MAO-A levels, the enzyme responsible for the degradation of 5-HT, adrenaline, noradrenaline, and dopamine in the CNS [100]. Second, estrogen inhibits the serotonin reuptake transporter (SERT) gene. The antagonistic effect of estrogen on SERT prolongs the action of 5-HT, leaving it in the synaptic space and synapses [101]. Estrogen also modulates the activity of 5-HT receptors. ERβ disruption upregulates 5-HT2A but not 5-HT1A protein expression in the female hippocampus [102].

## Estrogen and inflammation

During menopause, a deficiency of estrogen aggravates immune disorders. Increased inflammatory reactions and autoimmune diseases are common in perimenopausal women [103]. Estrogen modulates the immune system through its anti-inflammatory action. Most immune and epithelial cells express ER [104]. 17β-estradiol protects the epithelium by modulating the expression of inflammatory cytokines and antimicrobial proteins [105]. The ERβ receptor regulates the innate immune response depending on the type of cellular environment. It may be a systemic pro-inflammatory process related to the activity of the HPA and the nervous system, including the sympathetic system. These systems protect the brain against ischemia, inhibit the activation of pro-inflammatory kinases, and stimulate mitochondrial functions [103].

Intracellular protein complexes (inflammasomes) are responsible for the inflammatory process by activating pro-inflammatory cytokines IL-1, IL-6, IL-18, and TNFα, TNFβ [106]. Decreased estrogen levels increase the cellular reactivity of these cytokines, increasing the number of cytokine receptors [107]. Elevated levels of IL-6 (a cytokine that stimulates inflammatory processes) underlie age-related disorders, including menopause and AD. IL-6 is also responsible for bone resorption and osteoclast increase leading to bone loss and osteoporosis during menopause [108]. In addition, it contributes to the development of cardiovascular diseases and diabetes, which can also be the basis of depressive disorders [109]. Administration of IL-6 is directly into the ventricles of the rat brain-induced depression-like behavior [110].

Increased levels of IL-18, a potent IFNγ-stimulating cytokine that elicits a robust inflammatory response found in many autoimmune and inflammatory diseases occurring in the post-menopausal period, are not evident before menopause [111, 112], showed a significant increase in IL-1β, IL-8, and TNFα in women with naturally occurring menopause compared to women of reproductive age without chronic inflammatory diseases. In rodents, TNFα causes behavioral disturbances, including decreased locomotor activity, changes in social behavior, and anhedonia [113]. 17β-estradiol serves a protective role in nervous system. Its deficiency increases inflammation by increasing the expression of molecules that promote inflammation of the nervous system. Elevated levels of pro-inflammatory cytokines play an important role in the pathophysiology of depression [114].

## Estrogen and metabolic activity

It is believed that estrogen directly influences mitochondrial functions, thus regulating mitochondrial biogenesis, respiration, ATP production, and the level of reactive oxygen species [115]. E2 may act directly on the mitochondria through interactions with ERα and ERβ receptors in the mitochondria of the hypothalamus, cortex, and Hip (Fig. 4) [78]. Moreover, estradiol modulation is essential for the expression of numerous mitochondrial genes, and its deficiency leads to elevated oxidative stress and mitochondrial dysfunction, followed by brain aging, cognitive impairment, and neurodegenerative diseases (Fig. 4) [116–118]. Interestingly, the brain structures studied most extensively in depressive disorders also show the highest expression of mitochondrial estrogen receptors [119, 120]. The relationship between mitochondria and estradiol levels seems to be a two-way street. On the one hand, estradiol directly affects the activity and quality of mitochondria, which can directly respond to intracellular levels of this hormone [121]. On the other hand, enzymes, such as StAR, 3β-HSD, 17β-HSD, and aromatase, involved in the synthesis of E2 and progesterone are localized in mitochondria [122].

These enzymes are involved in steroid hormone (E2) production in the ovary and brain. Therefore, mitochondrial quality is crucial for synthesizing brain E2 [123]. Blood antioxidant levels decrease in patients after ovariectomy and the perimenopausal period [116, 124]. The loss of antioxidant homeostasis negatively affects the quality of mitochondria in many tissues, including the brain [125], and antioxidant production seems to depend on E2 levels. Recently, Zhao et al. [126] showed mitochondrial dysfunction and cognitive decline in a mouse model of ovariectomy-induced E2 deprivation.

Contrary to other studies, E2 supplementation can restore appropriate levels of antioxidant enzymes [116]. Notably, a decrease in blood antioxidant levels and mitochondrial dysfunction have been described as common problems in patients with depression [18, 127]. Thus, many recent studies point to the role of mitochondrial activity, decreased ATP production, and mitochondrial damage as significant factors involved in the development of depressive disorders [128–131]. Furthermore, it is believed that many of the previously described mechanisms of the etiology of depression, such as disturbed neurotransmitter release, maintenance of neurogenesis, and neuroplasticity, also depend on mitochondrial quality [18, 132, 133]. Some studies have shown that disturbances in the insulin signaling pathway may be associated with mitochondrial dysfunction, especially in conditions of low estrogen [134].

It should be noted that disturbances in the insulin signaling pathway are thought to contribute to depressive disorders [135]. In conclusion, there seems to be a link between E2 levels, mitochondrial quality, and depressive disorders. Therefore, it is suggested that depressive disorders in the perimenopausal period may also be associated with the loss of mitochondrial function in the brain.

## Conclusion

Menopause, as a naturally occurring physiological phenomenon, reflects persistent hormonal changes, including decreased estrogen production from the ovarian follicles. Symptoms associated with menopause, especially in the preceding perimenopausal period, include genitourinary disorders, cardiovascular disorders, musculoskeletal pain, hot flashes, night sweats, and changes in mood and cognitive functions. Both mood and cognition have and continue to attract attention in clinical diagnosis and experimental studies and form the bases for detailed studies of brain structure changes and neurotransmission during menopause. While these studies hold some truths, data from studies involving women present some ambiguities. Models involving menopause cannot be "standardized" due to different environmental conditions/stressors, the length of reproductive age in women, lifestyle and health status, and even ethnic differences. There are also differences in patient selection criteria in published works.

The arguments showing the relationship between the menopausal period and the onset of depressive disorders include the presence of intense hormonal fluctuations in periods, such as before menstruation, during pregnancy, and puerperium, and during the perimenopausal period. Additionally, estrogen exerts a strong neuroprotective effect through receptors in numerous brain structures. Polymorphisms in these receptors have been documented in late-onset depression.

Furthermore, the monoamine neurotransmitter systems involved in the etiology of depression are regulated by estrogen. Estrogen therapy, in turn, can prevent the emergence of depressive symptoms in perimenopausal and early post-menopausal women [136].

The above-described mechanisms, including the unmistakable influence of estrogen on pathways involved in neuroplasticity, neurotransmission, inflammatory responses, and metabolic activity, have been extensively described in studies using animal models of menopause. Further, especially clinical studies are warranted to provide helpful information in the diagnosis and treatment of depression, especially in menopausal women.

## Data Availability

Data sharing does not apply to this article as no data sets were generated or analyzed during the current study.

## Abbreviations

3β, 17β-HSD: 3β,17β-hydroxysteroid dehydrogenase
5-HT: Serotonin
AD: Alzheimer's disease
ADs: Antidepressants
Amy: Amygdala
ATP: Adenosine triphosphate
BDNF: Brain-derived neurotrophic factor
Cb: Cerebellum
CNS: Central nervous system
CREB: Cyclic AMP-responsive element binding
E2: Estradiol
ER: Estrogen receptors
FSH: Follicle-stimulating hormone
GDNF: Glial cell-derived neurotrophic factor
GnRH: Gonadotropin-releasing hormone
GPER, Gq, ERα, ERβ, ER-X: Membrane and cytoplasmic estrogen receptors
Hip: Hippocampus
HPA: Hypothalamic–pituitary–adrenal axis
IFNγ: Interferon γ
IL-1–18: Interleukin 1–18
LH: Luteinizing hormone
MAO: Monoamine oxidase inhibitors
NMDA: *N*-Methyl-d-aspartate receptor
NT-3: Neurotrophin-3
O&NS: Oxidative and nitrosative stress
PD: Parkinson's disease
PFC: Prefrontal cortex
PMDD: Premenstrual dysphoric disorder
POW: Premature ovarian failure
SERT: Serotonin reuptake transporter (SERT)
SNRI: Selective noradrenaline reuptake inhibitors
SSRI: Selective serotonin reuptake inhibitors
StAR: Steroidogenic acute regulatory protein
STRAW: Stages of Reproductive Aging Workshop
TNFα,β: Tumor necrosis factor α, β
WHO: World Health Organization
